# Striking a Balance: Socioeconomic Development and Conservation in Grassland through Community-Based Zoning

**DOI:** 10.1371/journal.pone.0028807

**Published:** 2011-12-21

**Authors:** Craig Leisher, Roy Brouwer, Timothy M. Boucher, Rogier Vogelij, W. R. Bainbridge, M. Sanjayan

**Affiliations:** 1 Central Science, The Nature Conservancy, Arlington, Virginia, United States of America; 2 Department of Environmental Economics, Institute for Environmental Studies, VU University, Amsterdam, The Netherlands; 3 Umgano Project Advisor, Umgano, KwaZulu-Natal, South Africa; University of California, Berkeley, United States of America

## Abstract

The goal of preserving nature is often in conflict with economic development and the aspirations of the rural poor. Nowhere is this more striking than in native grasslands, which have been extensively converted until a mere fraction of their original extent remains. This is not surprising; grasslands flourish in places coveted by humans, primed for agriculture, plantations, and settlements that nearly always trump conservation efforts. The Umgano grassland conservation and poverty reduction project in KwaZulu-Natal Province, South Africa uses community-based spatial planning to balance the conversion of its lower-conservation value grasslands to a timber plantation, while conserving higher-value grasslands for heritage purposes and managed livestock grazing. Ten years after project launch, we measured the ecological and socioeconomic impacts of the project using Normalized Differential Vegetation Index remote sensing data and over 500 household interviews, as compared with similar non-conserved areas. Zoned management of the Umgano area had resulted in between 9% and 17% greater average peak production in the grassland areas compared to control sites. There was also a 21% gain in incomes for the roughly one hundred people employed by the forestry efforts, when compared to others in their village. Community-based spatial zoning is an overlooked tool for balancing conservation and development but may require, as we found in Umgano, certain critical factors including strong local leadership, an accountable financial management mechanism to distribute income, outside technical expertise for the zoning design, and community support.

## Introduction

Grassland ecosystems support livelihoods for nearly 800 million people worldwide, providing livestock forage, wildlife habitat, and a host of other resources [1]. However, it is estimated that three quarters of the world's grazing lands have become so degraded that they have lost at least a quarter of their capacity to support animals [2]. In South Africa, grasslands and savannas provide livelihoods for people and habitat for many threatened and endemic species but face increasing risk of transformation into pastures, farmland and timber plantations [3]-[5]. Among the WWF global 200 ecoregions, South African montane grasslands are listed as critically endangered [6]. Only 1.6% of the grasslands in South Africa are formally protected, and fragmentation of grasslands by commercial timber plantations is of particular concern [7]. Since 2004, approximately 11,500 km^2^ of grasslands (about 3.3% nationally) have been cleared and planted with non-native *Eucalyptus* spp. and *Pinus* spp. [7].

Afforestation poses a special threat to South African grasslands because the areas of highest grassland biodiversity largely overlap with the areas most suitable for timber plantations [7]. However, it is not just afforestation that poses a threat. Expanding agriculture, fencing of rangelands, and climate change are impacting grasslands and pastoral livelihoods [5]. Grasslands support some of Africa's highest concentrations of livestock, and poor farmers often have little choice but to cultivate crops and graze cattle on marginal lands, which can lead to a cycle of increasing soil erosion and land degradation [1], [8].

African grazing systems have generally been common-pool resource management systems, which recognize legitimate users and regulate access by outsiders through sophisticated mechanisms of negotiating exclusion and enforcement [9]. However, where such traditional resource management systems are not in place, grassland management is frequently confronted with over-grazing, leading to deterioration of vegetation, increased soil erosion, and lowered biodiversity [10]. This in turn may result in general rangeland deterioration and decreased animal production [11]. Traditional management systems may also be undermined by a local desire to create new economic opportunities in the grasslands by changing land-use practices.

Here, we present an example of a South African grassland in which a local community facing the above-mentioned pressures used careful spatial zoning of community grazing land to achieve both grassland conservation and socioeconomic development. Spatial zoning is considered a key tool for administration, management, and conflict resolution in places as diverse as urban neighborhoods and protected areas [12], [13]. Zoning inherently requires the evaluation and trade-off of multiple objectives [14], [15]. In this study, 20% of the study area with lower conservation-value grasslands was used to support local socioeconomic development and generate funds for the preservation of the higher conservation-value grasslands in the rest of the area. In the context of conservation, the complexity of such tradeoffs has spawned computer software to analyze relevant variables and support decision-making [16]. Yet spatial zoning need not be complex to be effective. Here we show that effective zoning can be simple, but that several factors may be critical to community success.

Ten years after the community zoning, the socioeconomic and ecological impacts are evaluated with the help of a combination of expert interviews, a grassland quality indicator based on ten years of remote sensing data, and a rural household survey. The aim of this article is to assess the success of this community-based conservation initiative, draw lessons for future conservation efforts, and contribute to the international debate and empirical evidence base regarding the relationship between biodiversity conservation and socioeconomic development [17]-[19].

### Umgano project

The project area subject to zoning covers 7,000 ha and is referred to as the Umgano Project Area (UPA). The UPA is located in the Umzikhulu District of southwestern KwaZulu-Natal Province in the foothills of the Drakensberg Mountain Range between 1,000 and 2,050 m above sea level. It lies in the summer rainfall area, with annual precipitation of 800 to 1,000 mm per annum. The principal vegetation types are Southern Mistbelt Forest, Drakensberg Foothill Moist Grassland, and Southern KZN Moist Grasslands. The UPA provides crucial habitat for a number of threatened and endemic avian fauna.

The area is state owned but under the jurisdiction of the Mabandla Traditional Council (MTC), which holds official custodianship rights. The Mabandla area has a population of about 22,000 people and is one of the poorest parts of the country, with no connection to the national electricity grid and no formal drinking water supply. Livestock is of great significance, not only as a source of income, but also as a sign of wealth and power [20]. Maximizing the number of cattle is a risk minimization strategy due to cattle theft and the die-off of livestock in winter.

Most of the UPA was previously freehold farming land that was expropriated in 1960 by the now-defunct Transkei Government and is technically State land on lease to the MTC from the Department of Rural Development and Land Reform. It may, therefore, be employed for any purposes acceptable to both the people of Mabandla and the State.

The MTC is the official traditional authority of the Mabandla people and is led by an hereditary chief. The Umgano Project was formed in 1998 as the result of a contract signed between the MTC and Mondi Forests, a commercial forestry company, to develop part of the area as a timber plantation. Mondi Forests, through its community forestry development program, provided the funding and technical assistance to undertake the necessary environmental assessments and initial planning. In 1999, Mondi Forests changed its strategic focus away from community forestry, but the Umgano community forestry project continued with the support of the government and several former Mondi Forests staff.

The MTC applied with the formal support of approximately 80% of MTC households to the Department of Rural Development and Land Reform for grant funding to establish the timber plantation. The department imposed two conditions for funding: establishment of a community trust to control the development and disburse benefits equitably; and the appointment of a reputable forestry company to ensure that the plantation was established and operated in accordance with industry good practice.

The MTC subsequently established the Mabandla Community Trust (referred to as ‘the Trust’) in 2000. The structure of the Trust comprises 12 trustees elected for four-year terms by each of the Mabandla administrative wards and an elected chairman. At the same time, MTC also established a subsidiary of the Trust, the Mabandla Development Company, which was responsible for the control of all technical, financial and business management undertaken within the UPA.

A Management Steering Committee advises on all activities that take place within the UPA and comprises the hereditary chief, the 12 trustees and trust chairman, professional advisors, as well as representatives of Ezemvelo KwaZulu-Natal Wildlife, the Department of Agriculture, Environmental Affairs and Rural Development, the Umzimkhulu Local Municipality, and the Grasslands Program of the South African National Biodiversity Institute.

Initial funding for the project came from a loan by Mondi Forests. In 2001, the Department of Rural Development and Land Affairs provided the requested grant for the development of the timber plantation of US$1.7 million equivalent, which was invested in the South African Land Bank and used as security for a loan from the Land Bank for a further US$300,000 equivalent, giving a working capital of US$2 million equivalent. These funds were used for establishing a Forest Stewardship Council-certified timber plantation of approximately 1,300 ha together with its associated infrastructure and paying back the Mondi Forests loan.

The project then took on new dimensions, through grants from the UNEP Climate Action Program grant to promote biodiversity conservation, employed *inter alia* for the training and mentoring of the field rangers, and from the EU-funded provincial Gijima Fund to enable the community and advisors to plan for an expanded Umgano Project. This grant resulted in an Integrated Management Plan that guides activities within the UPA [21] and builds on the zoning proposed in the initial environment impact analysis [22].

The Integrated Management Plan divides the UPA into three zones: a biodiversity conservation zone of approximately 1,300 ha; a commercial afforestation zone of 1,500 ha; and a livestock management zone of 4,200 ha. The biodiversity conservation zone is in the process of being formally designated as a nature reserve to be co-managed by the community and the provincial wildlife authority Ezemvelo KwaZulu-Natal Wildlife [23].

By 2010, the number of employees of the Mabandla Development Company had grown to two supervisors and approximately 60 permanent and a further 40 non-permanent staff, all recruited from the Mabandla community. Nothing comparable to the Umgano Project can be found in communities that neighbor Mabandla. The annual revenue from the plantation was close to USD 240,000 equivalent in 2010, allowing continued investments in the socioeconomic development of the Mabandla area. The revenues from the plantation support community projects, including conservation of biodiversity in the conservation zone and a local health clinic. The revenues also fund the administration costs of the designated nature reserve and field rangers who patrol the project area under the authority of the MTC. Community benefits are expected to increase further as the plantation matures.

## Methods

Monitoring the ecological and social impact of community-based approaches to common-pool resource management is rare, despite calls from conservationists over the past decade [24]-[26]. Typically, much of the existing empirical evidence on the relationship between socioeconomic development and conservation is based on qualitative case-study narratives instead of well-designed monitoring studies [27].

To demonstrate the impact of a conservation project in a statistically robust manner, one can either do a ‘before-after’ project implementation comparison (at different points in time) or ‘inside-outside’ project area comparison (at the same point in time) or both [28]. We assessed the ecological and social impacts of the Umgano project using an inside-outside approach because of the absence of any baseline data.

The ecological assessment draws on time-series data analysis using the Moderate Resolution Imaging Spectroradiometer (MODIS) sensor based on Normalized Differential Vegetation Index (NDVI) as a measure of photosynthetic activity inside and outside the UPA. MODIS NDVI has demonstrated the ability to estimate total and live biomass, and can reliably detect the phenology and forage quantity and quality of grassland steppe areas [29], [30]. NDVI has also been used to study anthropogenic effects on grasslands, including overgrazing [31]-[33] and restoration efforts [34].

In order to assess the impact of ten years of zoning on grassland quality, sites within the UPA were paired with control sites outside the project area. One lowland (<1,400 m) in the grazing zone and one highland (>1,400) site in the conservation zone were randomly selected within the UPA, and each was matched with four control sites of similar elevation (within 100 m), similar rainfall as per NASA Monthly Global Precipitation data (within 100 mm), similar land use (e.g., no trees and similar grass to surrounding areas), and similar soil types. In the absence of detailed soil maps, vegetation types was used as a coarse proxy for soil type, using Drakensberg Foothill Moist Grasslands for the highlands sites and Southern KwaZulu-Natal Moist Grasslands for the lowland sites. Care was taken not to include Southern Mistbelt Forest in any of the sample sites. The sample sites were on low to moderate slopes with varying aspects within each site. An attempt was made to locate the sample sites in areas with similar household densities, but with no *a priori* data, this was estimated in the field. All sample sites were field checked to ensure treated and control sites fit the matching criteria.

The highland treated site was also compared with a nearby fully protected highland site within a nature reserve using the same matching criteria.

Each sample site was 5.0625 km^2^. MODIS pixels are 250 m×250 m, and a 9×9 pixel set (2.25 km×2.25 km) was analyzed to maximize the signal to noise ratio. The NDVI data were daily samples averaged into 16-day blocks by NASA and the Oakridge National Laboratory of the US Government. Cloud-covered pixels were excluded from the 16-day averages. No sub-pixel interpolation was needed.

Summarized NDVI statistics were gathered over a 10.5-year time-span (January 2000 to June 2010) and used to compare the habitat condition of conservation sites against field-calibrated control sites. The statistics were summarized for both the conservation area and the control sites to produce average seasonal NDVI characteristics and a Time-Integrated NDVI, i.e., summed growth season NDVI (September to April) of all 81 pixels for each site. This statistic is strongly correlated with above-ground biomass [35], [36].

**Figure 1:**
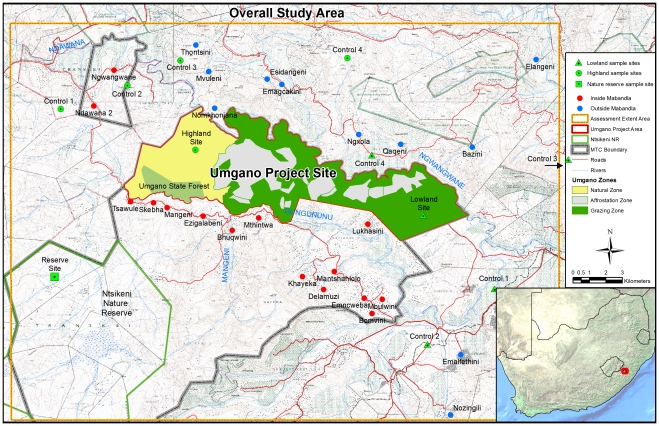
Location of villages included in the rural household survey and ecological sampling sites.

We assessed the socioeconomic impacts of the project via a rural household survey administered both inside and outside the MTC region (Figure 1). Inside the MTC, 15 villages were selected at a range of distances from the UPA. In these villages, 376 face-to-face household interviews were conducted using a structured household questionnaire. Outside the MTC, 140 household interviews were carried out in 12 villages in neighboring communities to the north and to the south. The traditional governance structure of Mabandla is the same as elsewhere in the province, with the presence of the UPA the only major distinction. Households were sampled randomly, in the absence of *a priori* socioeconomic census information.

The household survey questionnaire consisted of four main parts. The first contained questions about the household's socio-demographic characteristics, including household composition, age structure, and education level. The second part addressed livelihood strategies, including sources of income, assets and dependency on natural resources such as water and fuel. This section also included information about household members' employment status, income from non-employment sources such as government grants and credit, and household assets, including housing characteristics, household appliances, and livestock. The third part recorded features of the household's location, including biophysical characteristics of the area and changes over the past 10 years, the structure of local governance and the extent to which respondents feel their interests are represented by the community leadership, and knowledge and perception of the advantages and disadvantages of the Umgano project. The final section of the questionnaire assessed cattle ownership, livestock holding conditions, and availability and location of grazing grounds over the past 10 years.

In the analysis, information about both monetary income and non-monetary income was used to assess household welfare and socioeconomic status [37]. In those cases where monetary income was used in the analysis, a distinction was made between productive income generated through active employment and total disposable household income including non-active employment sources such as government grants, which made up a substantial share of total household monetary income. Different income information formats were used to prompt truth telling, including cross-checks with regards to employment status of all household members, professional activities, and the number of months during the year people were employed fulltime or part-time.

The household questionnaire was developed in collaboration with local experts, translated into the local dialect, and pre-tested by five local enumerators who then conducted the household survey. The enumerators were selected from a group of educated villagers living outside the UPA between 21 and 35 years old who had no ties to the project. They were trained in interview techniques during a one-day workshop and subsequently involved in three rounds of pre-testing, as well as debriefed about the interview results each day during the survey by the field survey supervisor.

A stratified random sampling procedure was used to select households for interviews in the villages. Enumerators were instructed to interview an equal number of men and women across different age groups, and respondents had to be older than 18 years. Interviews lasted between 25 and 45 minutes. The questionnaires were checked before data entry by the field survey supervisor to ensure that they were complete, then translated and entered into a pre-structured Excel database using data validation functions to minimize entry errors. The Excel database was in turn converted into a SPSS database for data cleaning and analysis.

In addition, 26 qualitative semi-structured interviews with local leaders, the project consultants, government officials, and local leaders of activities funded by project revenues were conducted to supplement the results from the quantitative analysis.

### Ethics Statement

We obtained verbal consent from participants before conducting household surveys. During verbal consent, participants were informed about the survey, its purpose, and how the data would be utilized. Written consent from participants was not obtained because of low literacy in the survey sites, which meant that participants may not have fully understood what they were signing. This study was managed by The Nature Conservancy, which does not have a formal Institutional Review Board, but the assessment plan was reviewed and approved by the senior levels of the organization. Formal permission for the research was codified in a Memorandum of Understanding with the The Nature Conservancy, the provincial government, and the Mabandla Traditional Council.

## Results

### Ecological assessment

**Figure 2:**
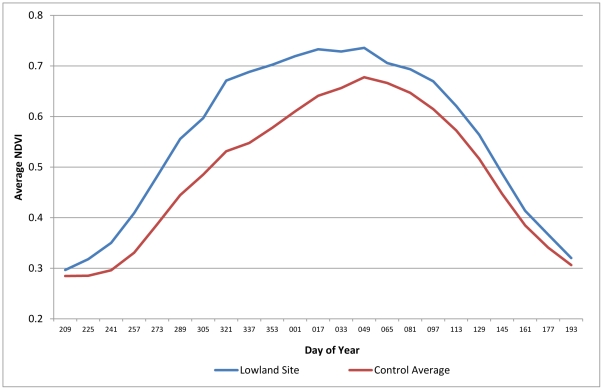
Lowland area, 10-year average seasonal NDVI. Shows the 10-year average NDVI by the day of the year, with higher NDVI equaling greater grass biomass, and earlier greening and later senesce showing a longer growing season. “Lowland controls” shows the average of the control sites.

**Figure 3:**
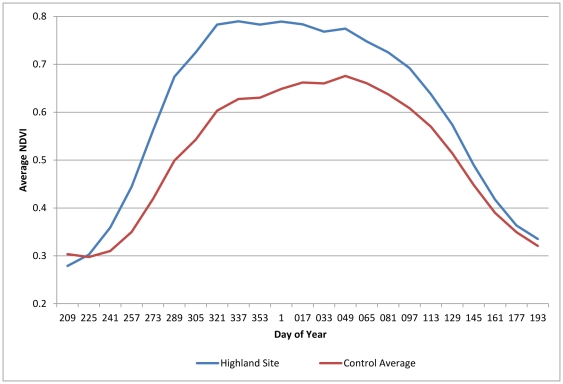
Highland area, 10-year average seasonal NDVI. Shows the 10-year average NDVI by the day of the year, with higher NDVI equaling greater grass biomass, and earlier greening and later senesce showing a longer growing season. “Highland controls” shows the average of the control sites.

**Figure 4:**
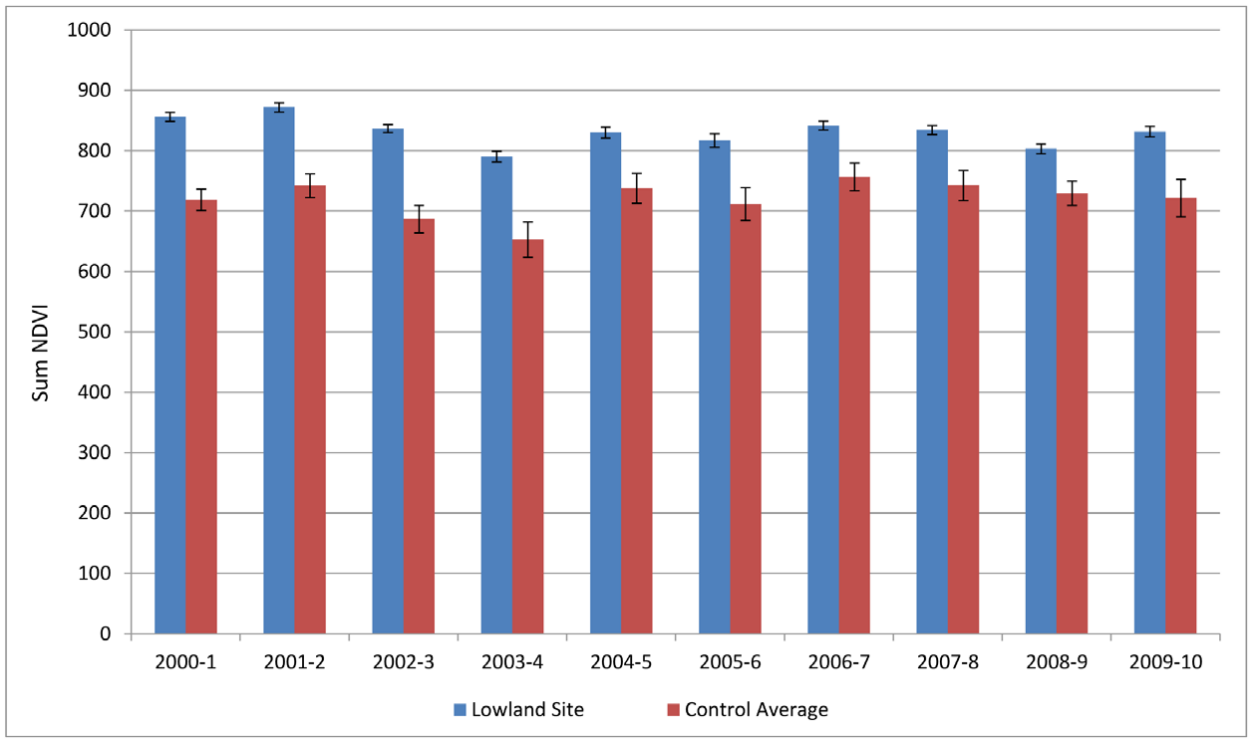
Lowland area, time-integrated NDVI per year. Shows the sum of the annual NDVI for each growing season from 2000 to 2010 which is a proxy for the total biomass produced in a site. “Lowland controls” shows the average of the control sites.

**Figure 5:**
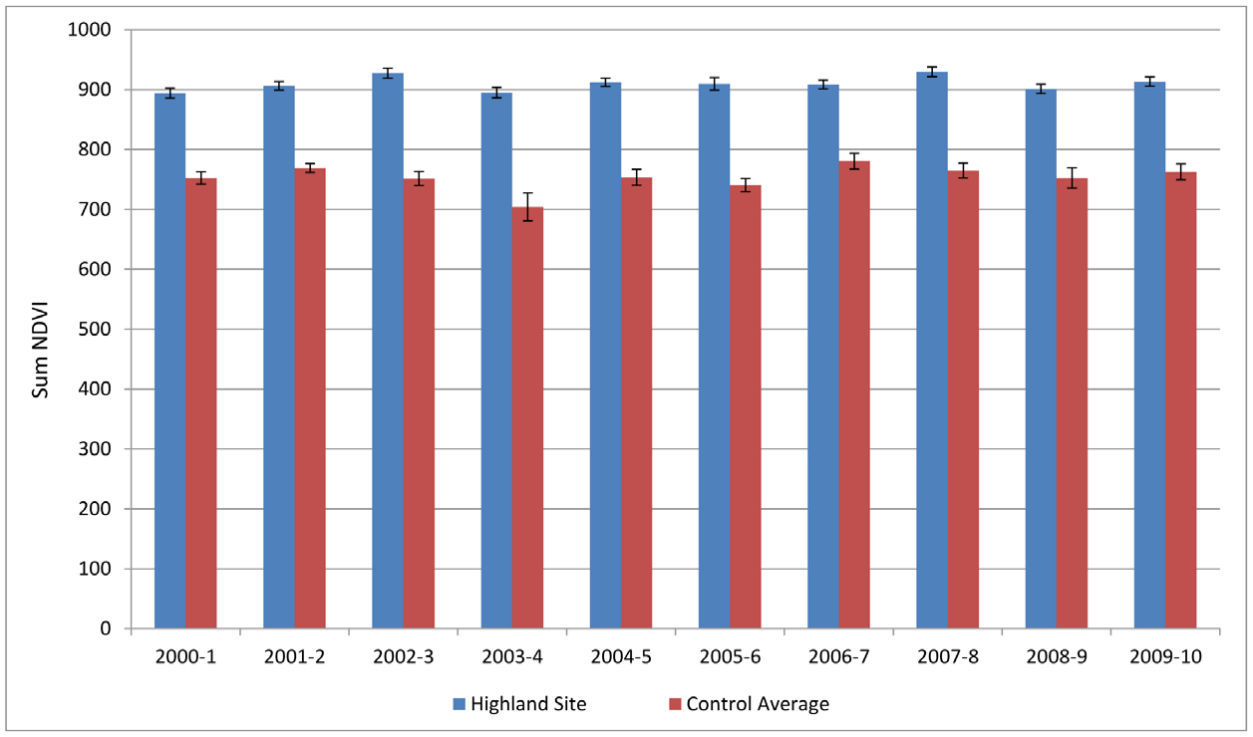
Highland area, time-integrated NDVI per year. Shows the sum of the annual NDVI for each growing season from 2000 to 2010 which is a proxy for the total biomass produced in a site. “Highland controls” shows the average of the control sites.

The ecological assessment shows that during the period of January 2000 to June 2010, the grasslands of the Umgano sites display faster and earlier recovery after winter senescence, and greater plant density, as shown by the higher NDVI values for summer growth. The average peak NDVI over a 10-year period was 9% higher in the Umgano lowland site (Mann-Whitney *p*<0.001) (Figure 2) and 17% higher in the Umgano highland site (Mann-Whitney *p*<0.001) (Figure 3) than in the corresponding control sites. The Umgano lowland site displayed more growth during the summer growing season and a slightly later onset of senesce in autumn than the four control sites. The highland site showed more rapid spring recovery than the four control sites. The Umgano sites also had consistently higher Time Integrated-NDVI and hence more biomass per year, with the lowland site averaging 15% higher than the control sites (Figure 4) and the highland site averaging 21% higher (Figure 5).

In comparing the Umgano highland site to the neighboring Ntsikeni Nature Reserve site (see Figure 1), we found that the Umgano site has a slightly earlier greening up and a slightly higher rate of growth but a similar maximum during the summer growth season. Overall, the two sites are quite similar, suggesting the grasslands in the Umgano project site are on par with a neighboring fully protected nature reserve.

### Socioeconomic assessment

Of the 513 households sampled across the MTC and the neighboring traditional councils, respondents were on average 45 years old, most were heads of their households or spouse of the head of the household, and ran a household of approximately six people of whom half were children. Respondents had an average of seven years of schooling, and about 15% of the sample population could not read or write. The most important energy sources were firewood (61%), followed by paraffin (26%), gas (5%) and electricity (5%). On average, a household spends four hours per day collecting firewood and one hour collecting water. Between 40% and 50% of the sample population is involved in crop farming, with maize being the most important staple crop, followed by potato. Very few households are fully self-sufficient year-round. In addition, 72% of all surveyed households take preventive soil erosion measures, primarily digging trenches and growing grass (Table 1).

**Table 1:** Sample household characteristics inside and outside the MTC.

	Inside MTC (*n* = 373)	St. Dev.	Outside MTC (*n* = 140)	St. Dev.
***Demographic characteristics***				
Share female respondents (%)	68.0		64.0	
Average age	45.0	17.3	47.4	16.9
Average household size	6.4	3.3	5.9	3.2
Average number of children	2.9	2.1	2.5	2.1
***Socioeconomic characteristics***				
Share illiterate (%)	14.4		13.8	
Average years of schooling	6.7	3.8	7.0	3.9
Average productive household income (ZAR/year)	10,113	30,840	10,504	26,942
Share depending on government grants only (%)	57.5		54.7	
Average total household income (ZAR/year)	17,176	31,264	20,313	37,831
Median total household income (ZAR/year)	9,000		8,640	
Average per capita income (ZAR/person/year)	3,827	14,648	3,992	8,815
Share under the international US$ 2 per day poverty threshold (%)	33.0		41.6	
Share borrowing money in community (%)	78.7		77.8	
***Housing characteristics***				
Share living in modern brick house (%)	33.2		48.8	
Share with electricity (%)	5.1		17.4	
Average amount of time to collect water (hours per day)	1.2	1.3	1.2	1.0
Share depending on forest for firewood and income (%)	27.3		17.1	
Average amount of time to collect firewood (hours per day)	4.1	2.0	3.7	1.8
***Livelihood characteristics***				
Share involved in crop farming (%)	39.8		50.3	
Share self-sufficient whole year round (%)	4.2		4.9	
Average number of months per year not self-sufficient	8.8	3.8	7.3	3.8
Share cattle holders in sample (%)	35.0		31.7	
Average number of cattle	9.0	9.7	8.4	6.5
Share suffering from soil erosion (%)	40.3		50.3	
Share taking soil erosion measures (%)	74.4		68.7	

Households within the MTC have significantly higher levels of total income (productive income and grant income taken together) than households from the two neighboring traditional councils (Kruskall-Wallis chi-square *p* = 0.024). MTC households also have greater per capita income (Kruskall-Wallis chi-square *p* = 0.024). Mean per capita income in the MTC is slightly higher than the international poverty line of USD 2 per day (or 3,810 ZAR), but 33% of sample households live under the international poverty line. This is comparable to the council to the south (27%) but substantially lower than the council to the north (53%).

It is difficult to establish a direct causal relationship between the Umgano project and improved socioeconomic conditions due to low levels of local awareness and recognition of the actions of the Umgano project. Indeed, only 19% of Mabandla respondents were able to identify effects of the Umgano project. Respondents were, therefore, asked whether their livelihood conditions had changed since the turn of the millennium. Perceptions of the benefits from the Umgano project were limited by this low level of awareness and recognition.

Perceptions of change in grassland accessibility and quality differed between households in the MTC and surrounding communities. Fewer than 8% of MTC livestock owners report having to travel further now than they did ten years ago to graze their stock, while outside the MTC this figure is 13%. Perhaps most importantly, 65% of stock owners outside the MTC said that the amount of bad grazing grass (known as “ngongoni” or wire grass, principally *Aristida junciformis*) had increased over the past 10 years compared to 36% inside the MTC. This suggests that MTC livestock owners have avoided a decline in grass quality.

The average number of cattle owned by a typical household (Table 2) as well as estimated household densities are similar inside and outside the MTC, suggesting that, *ceteris paribus*, stocking rates are unlikely to be an explanatory variable for this difference in perceived grass quality.

**Table 2:** Cattle holder characteristics inside and outside the MTC.

	Inside MTC (*n* = 140)	St. Dev.	Outside MTC (*n* = 51)	St. Dev.
***Demographic characteristics***				
Average age	48.0	18.0	46.5	16.2
Average household size	7.1	3.5	7.3	3.4
Average number of children	3.2	2.2	3.1	2.2
***Socioeconomic characteristics***				
Share illiterate (%)	17.7		4.0	
Average years of schooling	6.7	4.1	7.5	3.2
Average productive household income (ZAR/year)	18,853	46,168	18,618	38,590
Average total household income (ZAR/year)	27,296	45,763	24,448	41,044
Average per capita income (ZAR/person/year)	6,204	23,853	4,289	10,893
Share under the international US$ 2 per day poverty threshold (%)	24.1		37.3	
Share borrowing money in community (%)	79.5		77.5	
***Housing characteristics***				
Share living in modern brick house (%)	45.4		56.9	
Share with electricity (%)	6.1		23.5	
Average amount of time to collect water (hours per day)	1.3	1.6	1.2	1.0
Average amount of time to collect firewood (hours per day)	4.1	2.0	3.3	1.7
***Livelihood characteristics***				
Share involved in crop farming (%)	63.9		70.6	
Share self-sufficient whole year round (%)	7.0		10.2	
Average number of months per year not self-sufficient	7.6	3.8	5.8	3.7
Average number of cattle	8.5	9.1	8.4	6.5
Share able to find grazing ground all year round (%)	70.5		70.5	
Average travel distance to find grazing ground (km)	2.3	1.9	2.2	1.8
Share who buys fodder (%)	64.4		65.4	
Average share cattle dying in winter (%)	18.2	22.1	13.8	16.1
Share believing unpalatable grass has increased past 10 years (%)	36.0		65.2	
Share suffering from soil erosion (%)	54.7		51.0	
Share taking soil erosion measures (%)	84.0		78.6	

Employment by the timber plantation was expected to be a key quantifiable project benefit, and thus 25 of the plantation's permanent employees were interviewed about their socioeconomic conditions. The productive household income of plantation workers is, on average, 21% higher than that of other households living in the same villages (USD 1,352 versus USD 1,111 annually equivalent) (Mann-Whitney *p*<0.001). Moreover, the share of plantation worker households that receive a government grant is substantially lower (42%) than other households living in the same villages (77%). The percentage of households owning cattle is roughly the same for plantation worker households and other households (35%). However, plantation worker households own considerably more cattle on average (17.4) than other households (9.1) (Mann-Whitney *p* = 0.084). No significant differences between the two groups can be found for other socioeconomic indicators.

## Discussion

The ecological and socioeconomic results indicate that the Umgano initiative generated both grasslands conservation and socioeconomic development benefits. Grasslands in the UPA showed higher levels of biomass and longer productive growing seasons than grasslands in surrounding regions. Moreover, the Umgano highland area has a similar NDVI signature as a nearby fully protected nature reserve, suggesting that the grasslands in Umgano are intact. Household interview data also suggests that grasslands within the project area were healthier. In addition, households within the MTC had higher levels of total income and per capita income, and those associated with the timber plantations showed further benefits in terms of higher income and livestock ownership.

Household questionnaires and key informant interviews allowed us to identify several factors that help explain why the Umgano project has had a measure of success. First, the community-based spatial zoning of the project area was simple, with three clearly defined zones and basic rules for resource use in each. The limited number of zones, the clearly delineated boundaries of the zones, and the shared social norms of the community are factors that have helped avoid community conflict over local resource use [13], [38].

Second, the MTC chiefs had the vision to take a longer-term approach, the skills to resolve local conflicts, and the willingness to partner with people outside the community. This was critical to the project's genesis and sustainability, and dovetails with findings by others that strong local leadership is a crucial success factor in local resource management initiatives [38]-[40].

Third, the establishment of a community trust provided a financial mechanism to ensure accountability and professional management of fiduciary responsibilities and the distribution of benefits to the community. The community has received a number of grants and loans where the Mabandla Community Trust with its formal legal structures and competent financial management played a critical role in attracting funders.

Fourth, the MTC sought outside expertise for help with project financial management, nature conservation, grassland and livestock management, and tourism. Several of the project advisors have worked on the project since inception. Other studies have also noted the catalytic effect of outside expertise on a conservation initiative [39], [41].

The community commitment to starting the timber plantation, with 80% of the community formally supporting it, provided the assurances to those who initially funded the plantation that there was sufficient local support to ensure the project would have time to generate benefits. This echoes the findings of others regarding the importance of widespread community support for local conservation initiatives [42]-[45].

While only 19% of survey respondents could name a benefit of the project (though this may have been due to several changes in the project's name), and only about 3% of the Mabandla households have identifiable and direct financial benefits from the new jobs in the timber plantation, this does not appear to have been critical to the project's continued public support. Other studies of community-based natural resource management have found the opposite and emphasize the importance of public support [46]-[48]. This suggests that the support for the project's continuation comes mainly from the MTC leaders, making it vulnerable to changes in leadership. Improved communications about the community initiatives funded by project revenues, in combination with increases in revenues from the timber plantation and planned investments in local infrastructure, are expected to enhance long-term community support for the project. Although not many Mabandla respondents linked these benefits to the project directly, a reduction in cattle theft due to field rangers who patrol the project area, and better access to healthcare services from the project-funded health clinic contributed to an overall improvement of living conditions in the MTC.

Finally, this paper provides quantitative evidence that it is possible to conserve native grasslands while improving the socioeconomic situation and that zoning is a useful and powerful tool for helping strike this balance.

## References

[1] White R, Murray S, Rohweder M (2000). Pilot analysis of global ecosystems: Grassland ecosystems.

[2] UNEP (2005). One planet many people: Atlas of our changing environment.

[3] Reyers B, Fairbanks DHK, Van Jaarsveld AS, Thompson M (2001). Priority areas for the conservation of South African vegetation: a coarse-filter approach.. Diversity and Distributions.

[4] O'Connor TG (2005). Influence of land use on plant community composition and diversity in Highland Sourveld grassland in the southern Drakensberg, South Africa.. Journal of Applied Ecology.

[5] Eriksen SHE, Watson HK (2009). The dynamic context of southern African savannas: investigating emerging threats and opportunities to sustainability.. Environmental Science & Policy.

[6] Olson DM, Dinerstein E (1998). The global 200: A representation approach to conserving the earth's most biologically valuable ecoregions.. Conservation Biology.

[7] Lipsey MH (2010). Do ecological networks in South African commercial forests benefit grassland birds? A case study of a pine plantation in KwaZulu-Natal.. Agriculture, Ecosystems and Environment.

[8] Turner MD, Ayantunde AA, Patterson KP, Patterson ED (2011). Livelihood transitions and the changing nature of farmer-herder conflict in Sahelian West Africa.. Journal of Development Studies.

[9] Homewood KM (2004). Policy, environment and development in African rangelands.. Environmental Science and Policy.

[10] Sansom AL (1999). Upland vegetation management: The impacts of overstocking.. Water Science and Technology.

[11] Ebrahimi A, Miloti T, Hoffmann M (2010). A herbivore specific grazing capacity model accounting for spatio-temporal environmental variation: A tool for a more sustainable nature conversation and rangeland management.. Ecological Modeling.

[12] Shlay AB, Rossi PH (1981). Keeping up the neighborhood: Estimating net effects of zoning.. American Sociological Review.

[13] Sabatini MD, Verdiell A, Iglesias RMR, Vidal M (2007). A quantitative method for zoning of protected areas and its spatial ecological implications.. Journal of Environmental Management.

[14] Geneletti D, van Duren I (2008). Protected area zoning for conservation and use: A combination of spatial multicriteria and multiobjective evaluation.. Landscape and Urban Planning.

[15] Babaie-Kafaky S, Mataji A, Sani NA (2009). Ecological capability assessment for multiple-use in forest areas using GIS-based multiple criteria decision making approach.. American Journal of Environmental Sciences.

[16] Watts ME, Ball IR, Stewart RR, Klein CJ, Wilson K (2009). Marxan with Zones: software for optimal conservation based land- and sea-use zoning.. Env Mod Software.

[17] Roe D (2008). The origins and evolution of the conservation-poverty debate: a review of key literature, events and policy processes.. Oryx.

[18] de Sherbinin A (2008). Is poverty more acute near parks? An assessment of infant mortality rates around protected areas in developing countries.. Oryx.

[19] Andam KS, Ferraro PJ, Sims KRE, Healy A, Holland MB (2010). Protected areas reduced poverty in Costa Rica and Thailand.. PNAS.

[20] Skelcher B (2003). Apartheid and the removal of black spots from Lake Bhangazi in KwaZulu-Natal South Africa.. Journal of Black Studies.

[21] Bainbridge WR, Alletson D (2009). Integrated Management Plan: Umgano Community Project, KwaZulu-Natal, South Africa.

[22] Bainbridge WR (1997). Streamlined Environmental Impact Report, Hoha-Nsikeni Afforestation Proposal..

[23] EKZNW (2007). KZN Biodiversity Stewardship Programme: Site Assessment.

[24] Pullin AS, Knight TM (2001). Effectiveness in conservation practice: pointers from medicine and public health.. Conservation Biology.

[25] Brooks JS, Franzen MA, Holmes CM, Grote MN, Borgerhoff Mulder MM (2006). Development as a conservation tool: Evaluating ecological, economic, attitudinal, and behavioral outcomes. ystematic Review No. 20.

[26] Sutherland WJ, Adams WM, Aronson RB, Aveling R, Blackburn TM (2009). One hundred questions of importance to the conservation of global biological diversity.. Conservation Biology.

[27] Pullin AS, Knight TM, Stone DA, Charman K (2004). Do conservation managers use scientific evidence to support their decision-making?. Biological Conservation.

[28] Ravallion M, Schultz TP, Strauss J (2007). Evaluating anti-poverty programs.. Handbook of Development Economics.

[29] Zhang X, Friedl MA, Schaaf CB, Strahler AH, Hodges JCF (2003). Monitoring vegetation phenology using MODIS.. Remote Sensing of Environment.

[30] Kawamura K, Akiyama T, Yokota H, Tsutsumi M, Yasuda T (2005a). Quantifying grazing intensities using geographic information systems and satellite remote sensing in the Xilingol steppe region, Inner Mongolia, China.. Agriculture, Ecosystems & Environment.

[31] Wang J, Rich PM, Price KP, Kettle WD (2005). Relations between NDVI, grassland production, and crop yield in the Central Great Plains.. Geocarto International.

[32] Kawamura K, Akiyama T, Yokota H, Tsutsumi M, Yasuda T (2005b). Comparing MODIS vegetation indices with AVHRR NDVI for monitoring the forage quantity and quality in Inner Mongolia grassland, China.. Grassland Science.

[33] Morton CD, DeFries RS, Shimabukuro YE, Anderson LO, Del Bon Espírito-Santo F (2005). rapid assessment of annual deforestation in the Brazilian Amazon using MODIS data.. Earth Interactions.

[34] Wang J, Guo N, Wang X, Wang J, Zhang J (2008). The monitoring and effect evaluation of restoring grazing to grassland project in Maqu County of China based on EOS/MODIS data. Geoscience and Remote Sensing Symposium, 2008. IGARSS 2008.. IEEE International.

[35] Tucker CJ, Townshend JRG, Goff TE (1985). African land-cover classification using satellite data.. Science.

[36] Wessels KJ, Prince SD, Zambatis N, MacFadyen S, Frost PE (2006). Relationship between herbaceous biomass and 1-km2 Advanced Very High Resolution Radiometer (AVHRR) NDVI in Kruger National Park, South Africa.. International Journal of Remote Sensing.

[37] UNECE (2007). The Wye Group Handbook of Rural Households' Livelihood and Well-Being Statistics on Rural Development and Agriculture Household Income.

[38] Ostrom E (2009). A general framework for analyzing sustainability of social-ecological systems.. Science.

[39] Pollnac RB, Crawford BR, Gorospe MLG (2001). Discovering factors that influence the success of community-based marine protected areas in the Visayas, Philippines.. Ocean & Coastal Management.

[40] Gutierrez NL, Hilborn R, Defeo O (2011). Leadership, social capital and incentives promote successful fisheries.. Nature.

[41] Leisher C, Van Beukering P, Scherl LM (2007). Nature's investment bank: How marine protected areas contribute to poverty reduction.

[42] Western D, Wright MR (1994). Natural connections: Perspectives in community-based conservation.

[43] Agarwal A, Gibson C (1999). Enchantment and disenchantment: The role of community in natural resource conservation.. World Development.

[44] Ancrenaz M, Dabek L, O'Neil S (2007). The costs of exclusion: recognizing a role for local communities in biodiversity conservation.. PLoS Biol.

[45] Marshall GR (2009). Polycentricity, reciprocity, and farmer adoption of conservation practices under community-based governance.. Ecological Economics.

[46] Barrow E, Bergin P, Infield M, Lembuya P, Bissonette JA, Krausman PR (1995). Community conservation lessons from benefit sharing in East Africa.. Integrating people and wildlife for a sustainable future.

[47] Mehta J, Heinen J (2001). Does community-based conservation shape favorable attitudes among locals? An empirical study from Nepal.. Environmental Management.

[48] Spiteri A, Nepal SK (2006). Incentive-based conservation programs in developing countries: a review of some key issues and suggestions for improvements.. Environ Manage.

